# Invasive Group A *Streptococcus* Infection among Children, Rural Kenya

**DOI:** 10.3201/eid2202.151358

**Published:** 2016-02

**Authors:** Anna C. Seale, Mark R. Davies, Kirimi Anampiu, Susan C. Morpeth, Sammy Nyongesa, Salim Mwarumba, Pierre R. Smeesters, Androulla Efstratiou, Rosylene Karugutu, Neema Mturi, Thomas N. Williams, J. Anthony G. Scott, Samuel Kariuki, Gordon Dougan, James A. Berkley

**Affiliations:** KEMRI-Wellcome Trust Research Programme, Kilifi, Kenya (A.C. Seale, K. Anampiu, S.C. Morpeth, S. Nyongesa, S. Mwarumba, N. Mturi, T.N. Williams, J.A.G. Scott, J.A. Berkley);; University of Oxford, Oxford, UK (A.C. Seale, J.A. Berkley);; University of Melbourne at the Peter Doherty Institute for Infection and Immunity, Parkville, Victoria, Australia (M.R. Davies);; University of Queensland, Brisbane, Queensland, Australia (M.R. Davies);; The Wellcome Trust Sanger Institute, Cambridge, UK (M.R. Davies, S. Kariuki, G. Dougan);; London School of Hygiene and Tropical Medicine, London, UK (S.C. Morpeth, J.A.G. Scott);; Murdoch Children’s Research Institute, Melbourne, Victoria, Australia (P.R. Smeesters);; University of Melbourne, Melbourne (P.R. Smeesters);; Public Health England, London (A. Efstratiou);; Imperial College, London (A. Efstratiou, T.N. Williams);; The Kenya Medical Research Institute, Nairobi, Kenya (R. Karugutu, S. Kariuki)

**Keywords:** child, children, neonatal, infection, *Streptococcus pyogenes*, group A *Streptococcus*, GAS, children, Kenya, bacteria

## Abstract

To determine the extent of group A *Streptococcus* (GAS) infections in sub-Saharan Africa and the serotypes that cause disease, we analyzed surveillance data for 64,741 hospital admissions in Kilifi, Kenya, during 1998–2011. We evaluated incidence, clinical presentations, and *emm* types that cause invasive GAS infection. We detected 370 cases; of the 369 for which we had data, most were skin and soft tissue infections (70%), severe pneumonia (23%), and primary bacteremia (14%). Overall case-fatality risk was 12%. Incidence of invasive GAS infection was 0.6 cases/1,000 live births among neonates, 101/100,000 person-years among children <1 year of age, and 35/100,000 among children <5 years of age. Genome sequencing identified 88 *emm* types. GAS causes serious disease in children in rural Kenya, especially neonates, and the causative organisms have considerable genotypic diversity. Benefit from the most advanced GAS type–specific vaccines may be limited, and efforts must be directed to protect against disease in regions of high incidence.

## Abstract 2

These infections cause serious illness, especially in neonates.

Worldwide, childhood deaths have decreased, largely attributable to fewer deaths from pneumonia, measles, and diarrhea (*1*); some of these reductions have been achieved through vaccination against common bacterial pathogens such as *Streptococcus pneumoniae* and *Haemophilus influenzae* type b (*2*). However, progress in reducing deaths among children has been slower in sub-Saharan Africa, where approximately half of all such deaths occur, a third during the first month of life (*1*). To achieve further disease reductions, it is essential to address other, potentially preventable, causes of invasive bacterial disease, such as group A *Streptococcus* (GAS). It is estimated that >660,000 cases of invasive GAS infection occur each year; >95% cases occur in resource-poor regions, and >160,000 patients die (*3*). Despite these estimates, data on invasive GAS infections in resource-poor settings are limited.

The Young Infant Study of invasive bacterial disease conducted in the late 1990s in The Gambia, Ethiopia, Papua New Guinea, and the Philippines reported GAS in 29 (17%) of 167 bacterial isolates from blood cultures and in 3 (7.5%) of 40 cerebrospinal fluid (CSF) cultures (*4*). Although this finding meant that GAS was the third most commonly isolated bacterium after *S. pneumoniae* and *Staphylococcus aureus*, research into associated invasive GAS infections has been limited. To our knowledge, in sub-Saharan Africa, only 1 estimate of invasive GAS incidence has been published: 29 cases/100,000 person-years (definite cases of bacteremia only) among children <5 years of age in Kenya and 96 cases/100,000 person-years among children <1 year of age (*5*). These incidences are higher than those reported from other resource-poor settings. Data from Fiji, in the Pacific, report an incidence of 26 cases/100,000 person-years among children <5 years and 45 cases/100,000 person-years among children <1 year of age (*6*). In New Caledonia, the incidence for children <5 years of age was 7 cases/100,000 person-years (*7*).

Vaccines for GAS are being developed; the most advanced is a 30-valent serotype-specific vaccine. Data about the *emm* types causing invasive GAS disease in sub-Saharan Africa are critical for assessing potential vaccine serotype coverage. Through comprehensive prospective clinical and microbiological surveillance (1998–2011), we determined incidence, clinical characteristics, and outcomes among children with invasive GAS infections in a hospital in rural Kenya. We used whole-genome sequencing to determine e*mm* types and phylogenetic variations of invasive GAS isolates. 

## Materials and Methods

### Study Design and Participants

Since 1998, the Kenya Medical Research Institute/Wellcome Trust Research Programme has undertaken prospective systematic clinical surveillance, including standardized clinical documentation and systematic microbiological investigation, for invasive bacterial disease among all children admitted for medical care to Kilifi County Hospital (in Kilifi, a rural area of coastal Kenya), as described elsewhere (*5*,*8*). Our observational study identified cases of invasive GAS disease during this surveillance of all children admitted to Kilifi County Hospital from August 1, 1998, through December 31, 2011. The study size was determined by admissions during the study period. The study was approved by the National Ethics Committee, Nairobi, Kenya (ERC 2144), and the Oxford Tropical Research Ethics Committee (OXTREC 151–12).

The denominator population was determined by using the Kilifi Health and Demographic Surveillance System, which covers 891 km^2^ surrounding the hospital and in 2011 included ≈260,000 residents (*9*); household enumerations are performed quarterly. We calculated the population age structure at the midpoint of the study (mid-2004) and the total number of live births.

### Clinical Surveillance and Case Definitions

At the time of patient admission to the hospital, a standardized set of clinical symptoms and signs were recorded and prospectively entered into a database. At the time of patient discharge, outcome was recorded. Anthropometry for the presence of kwashiorkor (edematous malnutrition) was systematically undertaken at admission and used to define severe acute malnutrition (*10*). For all nonelective admissions, samples were collected for complete blood count, malaria slide, and blood culture. If clinically indicated, culture was performed for CSF, urine, and pus swab samples. Inpatient treatment was provided according to World Health Organization guidelines (*10*).

Starting in January 2007, in line with national guidelines, HIV testing by rapid test was offered for all children admitted. For children who had invasive GAS disease before 2007 and were not tested during our previous study of bacteremia (*5*), a trained counselor visited households and offered voluntary counseling and testing (*9*). For children who had died or were untraceable, a stored blood sample was tested by PCR for HIV. Sickle cell disease testing by electrophoresis was undertaken as clinically indicated; for children admitted with bacteremia during 1998–2008, PCR was used to retrospectively test for sickle cell disease, as described previously (*11*).

For this analysis, data were extracted from clinical and laboratory databases. All paper clinical records were reviewed for signs and symptoms relevant to invasive GAS disease, including the presence of pharyngitis, burns, scabies, and a vesicular rash suggestive of varicella or herpes zoster infection.

 Cases of invasive GAS were defined as definite if GAS was isolated from a normally sterile site (blood, CSF, or other sterile fluid/tissue) or if necrotizing fasciitis with evidence of GAS infection was present (e.g., typical gram-positive cocci found after Gram staining or serologic testing results positive for streptococci). Cases of invasive GAS were defined as probable if any of the following were found: classic necrotizing fasciitis without microbiological confirmation; cellulitis in a patient who was moderately or severely unwell (i.e., unwell and history of parenteral receipt of antimicrobial drugs, admission to hospital, or both); microbiological confirmation (i.e., growth of GAS on culture of swab sample or serologic test results positive for streptococci); or other clinically relevant infection in a patient who is moderately or severely unwell (i.e., unwell and history of parenteral receipt of antimicrobial drugs, admission to hospital, or both), in conjunction with positive GAS culture from deep wound swab sample or biopsy sample from surgical infection site (*6*).

Clinical syndromes of invasive GAS disease vary. These syndromes were categorized as meningitis, severe pneumonia, skin or soft tissue infection, joint and bone infection, necrotizing fasciitis, urinary tract infection, acute glomerulonephritis, abdominal disease, endocarditis, bacteremia with no focus, and streptococcal toxic shock syndrome (Technical Appendix Table 1).

### Microbiological and Molecular Methods

Blood cultures were undertaken by using the BACTEC Peds Plus system (Becton Dickinson, Franklin Lakes, NJ, USA) according to the manufacturers’ instructions. Positive broth cultures and CSF, urine, and surface swab samples were subcultured on 5% horse blood agar and chocolate agar. GAS isolates were identified by β-hemolysis, followed by Gram staining and catalase testing, and then grouped by latex bead agglutination. Penicillin susceptibility was tested by disk diffusion (http://www.bsac.org.uk/). Laboratory procedures were subject to internal quality control and external quality control by the UK National External Quality Assessment Service.

GAS isolates were subcultured on 5% horse blood agar from archived bacterial isolates (stored at −80°C) and transported to the Wellcome Trust Sanger Institute, Cambridge, UK. DNA was extracted by a QIAxtractor (QIAGEN, Valencia, CA, USA), and DNA quality and quantity were documented by using NanoDrop (Thermo Scientific, Waltham, MA, USA) and Qubit (Life Technologies, Carlsbad, CA, USA) techniques. Whole-genome sequences were determined from Illumina 96-plex libraries by using the HiSeq2000 sequencing platform (Illumina, San Diego, CA, USA) to generate tagged 75-bp paired-end reads. To obtain the overall population structure of the sequenced genomes, we mapped individual Illumina read pairs to the MGAS5005 (*emm*1) reference genome (*12*) by using SMALT version 0.5.8 (http://www.sanger.ac.uk/resources/software/smalt/). The average coverage of the resulting whole-genome alignment was 190×. The minimum base-call quality for identifying a single nucleotide polymorphism (SNP) was set at 50, and the minimum mapping quality for SNP calling was set at 30. SNPs called in known MGAS5005 prophage regions and repeat regions were excluded from analyses. The final genome alignment was 1,629,062 bp and comprised 125,233 SNPs. To examine the genomic relationships between the sequenced genomes, we generated a maximum-likelihood tree from the SNP alignment by using FastTree (*13*). Draft genome assemblies were compiled by using an iterative sequence assembly process as defined previously (*14*). An initial quality control screen of the short-read sequences to identify mixed isolates and low-quality sequences was determined by examining genome assembly length and SNP heterogeneity. A total of 43 (11.6%) sequences had an assembly length of >2 mega basepairs and were excluded from phylogenetic analyses because of possible contamination. The *emm* type and multilocus sequence type (MLST) were obtained from in-house BLAST analysis of draft genome assemblies and compared with those in centralized databases (http://www.cdc.gov/streplab/m-proteingene-typing.html, http://pubmlst.org/spyogenes/). New *emm* and MLST alleles were assigned by database curators. Allocation of *emm* cluster was derived as previously described (*7*). Heterogeneity observed within the typing schemes was investigated by using maximum-likelihood associations in whole-genome sequence data.

### Epidemiologic Analysis

Epidemiologic analyses were undertaken by using STATA version 13 statistical software (StataCorp LP, College Station, TX, USA). Clinical characteristics of children with invasive GAS disease were tabulated, and the frequency of clinical syndromes of invasive GAS disease and associated case-fatality risks (by age group) were calculated. Incidence rates were calculated by using the invasive GAS cases resident within the Kilifi Health and Demographic Surveillance System, the age structure of the population at the study mid-point (2004), and the total number of live births. Trends in admissions were examined by using rolling averages, and a comparison between seasons (wet and dry) was made by using the Poisson distribution.

## Results

During the study, 64,761 children were admitted to the hospital with acute illness. From 370 children with invasive GAS infection, 391 GAS isolates were identified. Of these 391 isolates, 154 (39.4%) were from blood, 9 (2.3%) from CSF, 214 (54.7%) from a swab sample (wound, skin breach, or pus), 8 (2.0%) from joint aspirates, and 6 (1.5%) from urine. From 20 children, >1 GAS isolate was identified: 7 children had invasive GAS isolated from both blood and CSF; 2 children had repeat positive blood cultures; 2 children had invasive GAS isolated from blood and a swab sample; 1 child had invasive GAS isolated from CSF and a swab sample; 7 children had invasive GAS isolated from 2 swab samples; and 1 child had invasive GAS isolated from 3 swab samples. No isolates were resistant to penicillin.

### Characteristics of Children and Risk Factors for Definite Invasive GAS Disease

Full clinical information was available for 369 of the 370 children: 152 children had definite and 217 had probable invasive GAS disease as defined. A total of 94 (25.5%) cases of invasive GAS were in neonates (Table 1). Among the 152 children with definite invasive GAS disease, 5 (3.3%) had burns, 4 (2.6%) had concurrent scabies, 1 (0.7%) had a vesicular rash (consistent with herpes zoster or varicella), and 2 (1.3%) had a history of trauma. Among the 217 with probable invasive GAS disease, 26 (12.0%) had burns, 3 (1.4%) had scabies, 1 (0.5%) had a vesicular rash, and 4 (1.8%) had a history of trauma (risk factors were not mutually exclusive). No reports of pharyngitis were documented for patients who had definite or probable invasive GAS disease. Among the 152 children with definite invasive GAS disease, prevalence of common risk factors for invasive bacterial disease was high: 81 (53.3%) had any risk factor; 30 (19.7%) had severe acute malnutrition, including 9 (5.9%) with kwashiorkor; 28 (18.4%) had malaria (slide positive for *Plasmodium falciparum*), and 24 (15.8%) had HIV infection.

**Table 1:** Characteristics of children with GAS disease admitted to Kilifi County Hospital, Kenya, 1998–2011*

Characteristic	All GAS disease, n = 369, no. (%)	Definite invasive GAS disease, n = 152, no. (%)	Probable invasive GAS disease, n = 217, no. (%)
Age			
0–6 d	33 (8.9)	13 (8.6)	20 (9.2)
7–28 d	61 (16.5)	38 (25.0)	23 (10.6)
29–59 d	17 (4.6)	12 (7.9)	5 (2.3)
60 d–1 y	63 (17.1)	40 (26.3)	23 (10.6)
>1 and <5 y	125 (33.9)	41 (27.0)	84 (38.7)
5–12 y	70 (19.0)	8 (5.3)	62 (28.6)
Sex			
M	219 (59.3)	84 (55.3)	135 (62.2)
F	150 (40.7)	68 (44.7)	82 (37.8)
Severe acute malnutrition			
No	294 (79.7)	106 (69.7)	188 (86.6)
Yes (wasting)	47 (12.7)	30 (19.7)	17 (7.8)
Yes (kwashiorkor)	11 (3.0)	9 (5.9)	2 (0.9)
Not known	17 (4.6)	7 (4.6)	10 (4.6)
Malaria (positive slide result)			
No	313 (84.8)	123 (80.9)	190 (87.6)
Yes	56 (15.2)	29 (19.1)	27 (12.4)
HIV infection			
No	209 (56.6)	116 (76.3)	93 (42.9)
Yes	28 (7.6)	24 (15.8)	4 (1.8)
Not known	132 (35.8)	12 (7.9)	120 (55.3)
Sickle cell disease			
No	136 (36.9)	95 (62.5)	41 (18.9)
Sickle cell trait	14 (3.8)	9 (5.9)	5 (2.3)
Sickle cell disease	3 (0.8)	1 (0.7)	2 (0.9)
Not known	216 (58.5)	47 (30.9)	169 (77.9)

*Malaria incidence (slide-positive admissions data from Kilifi Health and Demographic Surveillance System) decreased from 28.5 to 3.45 cases per 1,000 person-years during 1999–2007. HIV prevalence was 4.9% (routine antenatal screening, 2004–2007) with no evidence of a temporal trend. Sickle cell disease prevalence among infants in the Kilifi Health and Demographic Surveillance System (2006–2009) was 15% for genotypes HbAS and 1% with HbSS (*11*)**.** Severe acute malnutrition is referenced against World Health Organization population standards (Technical Appendix Table 1). GAS, group *A Streptococcus.*

### Clinical Syndromes of GAS Disease and Case-Fatality Risk

Among the 369 children with invasive GAS disease, the most frequent infection was skin or soft tissue infection, occurring in 258 (69.9%); followed by severe pneumonia in 86 (23.3%), of which 59 (69%) were complicated by sepsis; then bacteremia without focus in 53 (14.4%) (Table 2). Also among these 369 children, 17 (4.6%) had bone and joint infections, 11 (3.0%) had meningitis, 6 (1.6%) had a urinary tract infection, 2 (0.5%) had acute glomerulonephritis, 1 (0.3%) had endocarditis, 1 (0.3%) had nonspecific abdominal signs, and 1 (0.3%) had necrotizing fasciitis. A total of 19 (5.1%) cases met the criteria for streptococcal toxic shock syndrome (*15*). Of the 369 children, 45 (12.2%) died. The case-fatality risk was highest among those with severe pneumonia (20/86, 23.3%), followed by primary bacteremia (11/53, 20.8%) and meningitis (2/11, 18.2%). Pneumonia and primary bacteremia occurred most frequently among children <1 year of age. 

**Table 2:** Common clinical syndromes of GAS disease among children admitted to Kilifi County Hospital, Kenya, 1998–2011

**Clinical syndrome **	**Age**						
	0–6 d	7–28 d	29–59 d	60 d–1 y	>1–<5 y	5–12 y	Overall
**All cases**							
No. (%)	33 (100)	61 (100)	17 (100)	63 (100)	125 (100)	70 (100)	369 (100)
Deaths, CFR	10 (30.3)	23 (37.7)	1 (6.3)	7 (11.1)	14 (11.2)	1 (1.4)	45 (12.2)
**Skin and soft tissue infection**							
No. (%)	22 (66.7)	33 (54.1)	5 (29.4)	37 (58.7)	99 (79.2)	62 (88.6)	258 (69.9)
Deaths, CFR	6 (27.3)	4 (12.1)	0	1 (2.7)	6 (6.1)	1 (1.6)	17 (4.5)
**Severe pneumonia†**							
No. (%)	7 (21.2)	17 (27.9)	8 (47.1)	28 (44.4)	21 (16.8)	3 (4.3)	86 (23.3)
Deaths, CFR	2 (28.6)	5 (29.4)	0	5 (17.9)	8 (38.1)	0	20 (23.3)
**Primary bacteremia**							
No. (%)	8 (24.2)	17 (27.9)	3 (17.6)	9 (14.3)	13 (10.4)	3 (4.3)	53 (14.4)
Deaths, CFR	2 (25.0)	5 (29.4)	1 (33.3)	2 (22.2)	1 (7.7)	0	11 (20.8)

*CFR, case-fatality risk; GAS, group A *Streptococcus*. †59 of the 86 severe pneumonia cases were complicated by sepsis.

### Incidence of Invasive GAS Disease

The minimum incidence (cases/100,000 person-years) for definite and all (definite and probable) invasive GAS disease, respectively, among children <5 years of age was 17 (95% CI 14–21) and 35 (95% CI 30–40); among children <1 year of age, incidence was 59 (95% CI 45–74) and 101 (95% CI 83–121). Among neonates, incidence (cases/1,000 live births) for definite and all invasive GAS, respectively, was 0.3 (95% CI 0.2–0.4) and 0.6 (95% CI 0.4–0.7). The incidence of death was 0.1 (95% CI 0.1–0.2) deaths per 1,000 live births (Table 3). No trend was detected in the number of cases admitted over the study period (Technical Appendix Figure 1). Invasive GAS cases occurred less frequently during the dry months across all years (December–March, 26 cases/month) than during months of the short and long rains (April–October, 33 cases/month) (p = 0.029).

**Table 3:** Estimated minimum incidence of definite and probable invasive GAS disease and deaths associated with invasive GAS disease in the catchment area of Kilifi County Hospital, Kenya, 1998–2011*

Incidence†	Age group				
	Neonate, 0–27 d, n = 9,828‡	Infant, 28–59 d, n = 10,463‡	Infant, 2–11 mo, n = 92,070‡	Child 1–4 y, n = 453,857‡	Child 5–12 y, n = 730,512‡
Probable and definite invasive GAS disease incidence (95% CI)	631 (484–808)	105 (52–188)	43 (31–59)	19 (15–23)	6 (4–9)
Definite invasive GAS disease incidence (95% CI)	326 (223–459)	86 (39–163)	27 (18–40)	7 (5–10)	1 (0–1)
Death associated with all invasive GAS disease (95% CI)	163 (93–264)	10 (0–53)	5 (2–13)	2 (1–3)	0 (0–1)

*GAS, group *A Streptococcus*. †Per 100,000 person-years. ‡Population denominator in person-years.

### Molecular Epidemiology of GAS

**Figure 1:**
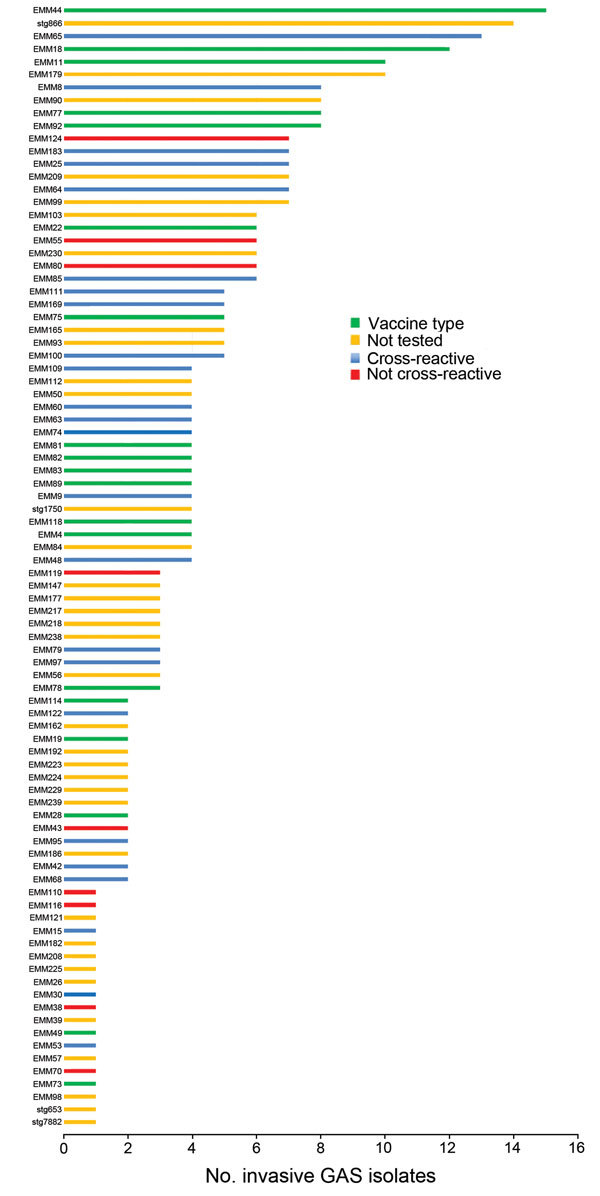
*emm* types of group A *Streptococcus* (GAS) isolates from children with GAS disease admitted to Kilifi County Hospital, Kenya, 1998–2011. *emm* types shown in green are included in the 30-valent vaccine; *emm* types in blue are not included in the 30-valent vaccine, but this vaccine may provide immunity to this *emm* type through cross-reactivity; *emm* types in red are not included in the 30-valent vaccine, and there is no evidence of cross-reactivity; *emm* types in yellow are not included in the 30-valent vaccine, and their cross-reactivity has not yet been tested.

Of the 391 original GAS isolates, we retrieved 371 and generated high-quality genome sequences for 328 (Technical Appendix Table 2). From another 29 GAS isolates (combined total of 357) with lower quality genome sequences, we were able allocate an *emm* type. The remaining 14 samples were subsequently excluded from molecular analyses because they were not GAS or were mixed cultures, affecting accurate SNP calling (but not epidemiologic analyses because these isolates had been subcultured, stored, and then subcultured again, potentially introducing contamination). Through BLAST analysis of the 357 genome sequences against the *emm* typing database, we assigned 88 different *emm* types (97 including subtypes). Of the *emm* subtypes, 21 were new variants. No *emm* types represented >5% of the isolates studied, showing that no single *emm* type was predominant in the GAS population irrespective of clinical association (Figure 1, Technical Appendix Figure 2).

**Figure 2:**
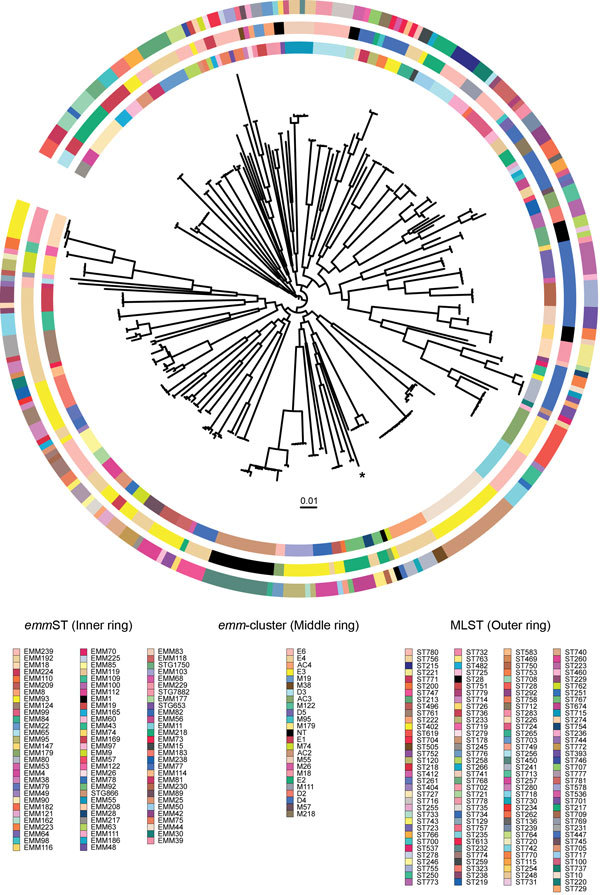
Population structure of 328 *Streptococcus pyogenes* strains from children with group A *Streptococcus* (GAS) disease admitted to Kilifi County Hospital, Kenya, 1998–2011*.* Unrooted maximum-likelihood phylogeny based on the whole-genome associations of mapped *S. pyogenes* genomes to the MGAS5005 reference genome indicates extensive genomic diversity within the population. The rings surrounding the central phylogeny correspond to standard GAS molecular typing methods; colors indicate different STs. Inner ring, *emm* ST (*16*); middle ring, *emm* cluster (*17*); outer ring, multilocus sequence type (*18*). NT, nontypeable *emm* clusters; ST, sequence type. *Position of the MGAS5005 reference genome. Scale bar indicates genetic change of 0.01.

Of the 357 GAS isolates, we assigned an *emm* cluster designation to 329 on the basis of the recently described *emm* cluster classification scheme (*16*). Of the 48 *emm* clusters described, 24 were represented within the Kilifi invasive GAS population of isolates (Technical Appendix Table 3). Of the 140 MLSTs identified, only 24 sequence types were represented within the MLST database (78/328 strains with high-quality whole-genome sequence data). We identified 89 new allelic variants among the 7 housekeeping genes and assigned 116 new MLSTs. Crude phylogenetic analyses of the Kilifi invasive GAS population as a whole revealed a star-like topology (Figure 2) indicative of diverse core genotypes. Collectively, these data illustrate substantial heterogeneity within invasive GAS genotypes in the Kilifi population.

In terms of vaccine coverage, 99 (28%) of 357 GAS isolates are included within the current 30-valent vaccine (*19*), and another 104 (29%) exhibit a degree of *emm* cross-reactivity in vitro (Figure 1) (*20*). Of the remainder, 27 (8%) were not included in the vaccine and are not cross-reactive, and 127 (36%) have not yet been investigated for cross-reactivity.

## Discussion

Incidence of invasive GAS disease in this rural sub-Saharan African setting was strikingly high, particularly among children in the first year of life among whom GAS was a major cause of sepsis and severe pneumonia. The minimum incidence of invasive GAS infection was highest among neonates (0.6 cases/1,000 live births; more than one third of all case-patients died). Minimum incidence in the first year of life overall was also high (101 cases/100,000 person-years), twice that for Fiji, the only other resource-poor setting from which an incidence estimate is available (*6*). The incidence estimates presented here are probably underestimates because inclusion in the study relied on hospital admission; hence, they are referred to as minimum incidence estimates. Residents living nearer to Kilifi County Hospital are more likely to access care than those living farther from it (*21*), and care-seeking behavior varies (*22*). The incidence of invasive GAS is probably accompanied by high prevalence of the spectrum of GAS infections, including acute poststreptococcal glomerulonephritis and acute rheumatic fever, which can lead to rheumatic heart disease (*23*); however, data for sub-Saharan Africa are limited (*24*,*25*).

In rural Kenya, unlike in other settings, pharyngitis, varicella, and scabies did not seem to be major drivers of invasive GAS disease (*23*,*26*), and impetigo was not differentiated from skin infections. These conditions are probably underascertained because they would not in themselves result in hospital admission, and unlike most of the clinical and microbiological data (systematically sought and collected), these diagnoses relied on observations being recorded. Also, despite the high frequency of skin and soft tissue infections, we detected only 1 case of necrotizing fasciitis, which may again be underascertainment from clinical information. Invasive GAS was, however, associated with concurrent conditions driving other bacterial diseases in sub-Saharan Africa: HIV, severe acute malnutrition, and malaria (*5*,*27*,*28*) but not sickle cell disease (as reported elsewhere) (*11*,*29*–*31*).

In this study, the invasive GAS *emm* types and *emm* clusters were extremely heterogeneous and differed from those that cause disease in resource-rich settings. The presence of several *S. dysgalactiae* subsp. *equisimilis*–like *emm* types within a *S. pyogenes* genomic backbone supports previous observations of interspecies genetic transfer of *emm* alleles (*32*). The overall diversity of *emm* types we describe supports findings of increased heterogeneity in other resource-poor settings (*33*). One published study reports noninvasive GAS *emm* types from sub-Saharan Africa. In that study, school children in Ethiopia were investigated for GAS carriage; 43 different *emm* types were identified in 82 colonizing GAS isolates (*34*). Less than one third of *emm* types identified in our study were also identified in the Ethiopia study, suggesting that the pool of GAS *emm* types in circulation, even within neighboring countries, is larger than that described here.

Reducing the incidence of invasive GAS infection in this setting could be achieved by reducing risk factors such as severe acute malnutrition and HIV (e.g., through prevention of mother-to-child transmission), as well as by supporting antisepsis measures at delivery, including antiseptic neonatal cord care (*35*–*37*). Early and improved treatment of skin infections, including impetigo, and burns could also reduce invasive GAS disease. However, prevention through effective vaccination will probably lower disease incidence the most, as has occurred for other pathogens, such as *S. pneumoniae* (*38*) and *H. influenzae* type b (*39*). The difficulty with *emm* type–specific GAS vaccine approaches (*19*) is the heterogeneity of GAS *emm* types and limited data on many of the *emm* types identified in this study. From current information, only 57% of invasive GAS disease cases would be covered (either directly or through cross-reactivity) by the most advanced 30-valent vaccine being developed (*19*). Furthermore, serotype replacement could occur, as described for *S. pneumoniae* (*40*), and would require detailed surveillance.

The high incidence of invasive GAS disease in rural sub-Saharan Africa underlines the contribution of invasive bacterial disease in this region to childhood deaths, particularly among neonates and young infants; associated case-fatality risk is high. Invasive GAS may also be causing puerperal sepsis in this setting; more studies are needed. Reductions in childhood illness and death could, however, be achieved through effective GAS vaccination. Further development of GAS vaccines followed by clinical trials must be prioritized, targeted at settings with the highest disease incidence.

Technical Appendix.  Invasive group A *Streptococcus *infection among children, Rural Kenya, 1998—2011. Definitions of clinical syndromes, details of *Streptococcus pyogenes* strains isolated, number of cases, and *emm* types of isolates.
