# Supplementary material for: Invasive Group A Streptococcus Infection among Children, Rural Kenya
Source: Emerg Infect Dis. 2016 Feb;22(2):224–32. doi: 10.3201/eid2202.151358 (PMC4734542; doi:10.3201/eid2202.151358)
Supplement: Supplementary file 1 — Technical Appendix. Invasive group A Streptococcus infection among children, Rural Kenya, 1998—2011. Definitions of clinical syndromes, details of Streptococcus pyogenes strains isolated, number of cases, and emm types of isolates. [file 15-1358-Techapp-s1.pdf]

Article DOI: http://dx.doi.org/10.3201/eid2202.151358

Invasive Group A Streptococcus Infection among Children, Rural Kenya

Technical Appendix

Table 1. Definitions of clinical syndromes

Clinical syndrome	Definition
Skin, soft tissue	Clinical observation (swelling, erythema, tenderness, redness) and positive GAS isolate from a clinical sample (swab or pus).
Bone and joint	Clinical observation (swelling, erythema, tenderness, redness) and/or positive GAS isolate from an aspirate.
Necrotizing fasciitis	Rapidly spreading infection of muscle fascia, fat and epidermis leading to necrosis.3
Severe Pneumonia	Severe pneumonia was defined according to WHO guidelines; cough or difficulty breathing plus at least one of central cyanosis or oxygen saturation <90%, severe respiratory distress, a general danger sign (inability to breastfeed or drink, lethargy or unconsciousness, convulsions),34 in a child with a positive isolate for GAS from a site of clinical infection.
Meningitis	Meningitis was defined by either a positive GAS culture from CSF, or CSF total leukocyte count ≥ 50 cells/μl and GAS isolated from another clinical site with signs of clinical infection.57
Bacteremia with no focus	Bacteremia with no focus was defined as a child with a positive GAS culture from blood, and no focus of infection (skin, soft tissue, bone and joint, pneumonia, meningitis, UTI, endocarditis or acute glomerulonephritis).
Urinary Tract Infection	Urinary tract infections were clinically defined (frequency or urgency) and the presence of a pure culture of GAS in a mid-stream urine sample.
Endocarditis	Documented evidence of a new heart murmur and positive blood culture for GAS
Acute glomerulonephritis	Hematuria with red cell casts on microscopy, and proteinuria
Streptococcal toxic shock syndrome	Adapted from the Working Group of Severe Streptococcal Infections,38 as isolation from a sterile site (definite case) plus hypotension (<5th percentile of systolic blood pressure in children) and two or more of the following: renal impairment (creatinine greater than twice the upper limit of normal for age), coagulopathy (platelets <100,000x106/l or evidence of disseminated intravascular coagulation), liver dysfunction (alanine transaminase, aspartate aminotransferase or bilirubin more than twice the upper limit of normal for age), adult respiratory distress syndrome (pulmonary infiltrates and hypoxemia without cardiac failure or generalized edema), generalized erythematous rash that may desquamate or soft tissue necrosis (necrotizing fasciitis, myositis or gangrene).
Severe acute malnutrition, SAM (subdivided into wasting or Kwashiorkor)	MUAC <11.0 cm (2 – 6 months) and <11.5cm in children 6 months or older;58 if MUAC was missing a weight for age Z score more than 3 standard deviations from the mean was included and for neonates a weight under 2500g. Kwashiorkor was defined by the presence of SAM with edema and wasting as SAM without edema.

Table 2. Details of S. pyogenes strains isolated from the Kilifi County Hospital (1998-2011) included in phylogenetic analyses

Name	Year	Patient age category	Specimen type	Clinical presentation	*emm* sequence type	*emm*-cluster designation+	MLST	Genome Sequence Accession no.*
K3525	1998	2–12 months	blood	sepsis (no localizing source)	EMM55.0	M55	ST100	ERR228579
K3534	1998	1–4 years	blood	skin, soft tissue	EMM65.0	E6	ST716	ERR439218
K3637	1998	1–4 years	swab	skin, soft tissue	EMM179.0	M179	ST619	ERR227079
K3573	1998	0–6 days	blood	sepsis (no localizing source)	EMM116.2	D4	ST702	ERR228576
K3589	1998	28–60 days	blood	meningitis, pneumonia	EMM8.3	E4	ST241	ERR439219
K3589	1998	28–60 days	blood	meningitis, pneumonia	EMM8.3	E4	ST241	ERR228640
K3730	1998	0–6 days	blood	sepsis (no localizing source)	EMM90.5	E2	ST708	ERR228598

Name	Year	Patient age category	Specimen type	Clinical presentation	*emm* sequence type	*emm*-cluster designation+	MLST	Genome Sequence Accession no.*
K3808	1998	7–27 days	swab	skin, soft tissue; pneumonia	EMM95.0	M95	ST712	ERR228655
K3828	1998	5 years and over	swab	skin, soft tissue; pneumonia	EMM238.1	A-C3	ST713	ERR228656
K3879	1998	1–4 years	blood	sepsis (no localizing source)	EMM183.2	E3	ST714	ERR228582
K3944	1998	1–4 years	blood	sepsis (no localizing source)	EMM122.0	M122	ST200	ERR228636
K3964	1999	7–27 days	swab	skin, soft tissue	EMM65.0	E6	ST716	ERR228657
K3997	1999	7–27 days	swab	skin, soft tissue	EMM65.0	E6	ST716	ERR228658
K4254	1999	2–12 months	blood	skin, soft tissue	EMM85.1	E6	ST774	ERR227080
K4134	1999	1–4 years	blood	skin, soft tissue	EMM209.1	E3	ST717	ERR228659
K4263	1999	1–4 years	swab	skin, soft tissue	EMM230.1	D4	ST755	ERR228661
K4240	1999	1–4 years	swab	skin, soft tissue; pneumonia	EMM74.0	M74	ST720	ERR228660
K4350	1999	28–60 days	blood	sepsis (no localizing source)	EMM22.5	E4	ST213	ERR228647
K4656	1999	7–27 days	swab	skin, soft tissue	EMM92.1	E2	ST727	ERR228662
K4728	1999	1–4 years	blood	pneumonia	EMM44.0	E3	ST178	ERR228594
K4761	1999	1–4 years	blood	pneumonia, stss	EMM8.3	E4	ST730	ERR228572
K4819	1999	2–12 months	swab	skin, soft tissue	EMM230.1	D4	ST755	ERR228663
K4828	1999	1–4 years	blood	sepsis (no localizing source)	EMM179.0	M179	ST619	ERR228689
K4982	1999	1–4 years	swab	skin, soft tissue	EMM124.2	E4	ST736	ERR228664
K4973	1999	7–27 days	blood	skin, soft tissue, pneumonia, stss	EMM25.4	E3	ST735	ERR228585
K4973	1999	7–27 days	blood	skin, soft tissue, pneumonia, stss	EMM25.4	E3	ST735	ERR228562
K5017	1999	2–11 months	blood	meningitis, pneumonia	EMM44.0	E3	ST178	ERR228575
K5017	1999	2–11 months	CSF	meningitis, pneumonia	EMM44.0	E3	ST178	ERR228696
K5086	1999	0–6 days	blood	sepsis (no localizing source), stss	EMM238.1	A-C3	ST713	ERR228555
K5248	1999	7–27 days	swab	skin, soft tissue	EMM43.1	D4	ST770	ERR227089
K5192	1999	1–4 years	blood	sepsis (no localizing source)	EMM147.0	NT	ST741	ERR228571
K5460	2000	0–6 days	swab	skin, soft tissue	EMM11.0	E6	ST742	ERR228665
K5499	2000	1–4 years	swab	skin, soft tissue	EMM65.5	E6	ST215	ERR439220
K5660	2000	1–4 years	swab	skin, soft tissue	STG866.1	NT	ST450	ERR228668
K5679	2000	1–4 years	blood	pneumonia	EMM177.0	E6	ST743	ERR228627
K5690	2000	0–6 days	swab	skin, soft tissue, pneumonia	EMM81.11	E6	ST744	ERR228669
K5698	2000	1–4 years	swab	skin, soft tissue	EMM109.1	E4	ST718	ERR227067
K5721	2000	7–27 days	swab	skin, soft tissue	EMM26.3	M26	ST745	ERR439221
K5727	2000	5 years and over	swab	skin, soft tissue	EMM25.1	E3	ST746	ERR228670
K5797	2000	0–6 days	swab	skin, soft tissue	EMM77.0	E4	ST747	ERR228673
K5797	2000	0–6 days	swab	skin, soft tissue	EMM77.0	E4	ST747	ERR227036
K5851	2000	7–27 days	blood	sepsis (no localizing source)	EMM230.1	D4	ST755	ERR228602
K5965	2000	1–4 years	swab	skin, soft tissue	EMM147.0	NT	ST753	ERR228676
K5898	2000	0–6 days	swab	skin, soft tissue	EMM209.1	E3	ST749	ERR228674
K5910	2000	5 years and over	swab	skin, soft tissue	EMM92.1	E2	ST750	ERR228675

Name	Year	Patient age category	Specimen type	Clinical presentation	*emm* sequence type	*emm*-cluster designation+	MLST	Genome Sequence Accession no.*
K5911	2000	7–27 days	blood	sepsis (no localizing source)	EMM217.0	D3	ST728	ERR439222
K6085	2000	5 years and over	swab	skin, soft tissue	EMM8.3	E4	ST241	ERR228680
K6008	2000	7–27 days	swab	skin, soft tissue	EMM75.1	E6	ST751	ERR228677
K6038	2000	7–27 days	swab	skin, soft tissue	EMM118.2	E3	ST752	ERR228678
K6048	2000	7–27 days	swab	skin, soft tissue	EMM147.0	NT	ST753	ERR228679
K6099	2000	0–6 days	swab	skin, soft tissue	EMM77.0	E4	ST482	ERR228681
K6102	2000	0–6 days	swab	skin, soft tissue	EMM109.1	E4	ST718	ERR227037
K6108	2000	2–11 months	swab	skin, soft tissue	EMM25.1	E3	ST323	ERR227078
K6222	2000	7–27 days	blood	skin, soft tissue	EMM70.0	D4	ST754	ERR228630
K6239	2000	2–11 months	blood	pneumonia, stss	EMM9.0	E3	ST740	ERR439224
K6363	2000	1–4 years	swab	skin, soft tissue	EMM230.1	D4	ST755	ERR228683
K6428	2000	1–4 years	swab	skin, soft tissue, pneumonia	EMM97.1	D5	ST756	ERR228684
K6429	2000	7–27 days	swab	skin, soft tissue	EMM38.1	M38	ST757	ERR439225
K7846	2001	7–27 days	blood	sepsis (no localizing source, stss	EMM74.0	M74	ST120	ERR228639
K6613	2000	1–4 years	blood	skin, soft tissue, pneumonia	EMM218.1	M218	ST292	ERR228702
K6635	2000	2–11 months	swab	skin, soft tissue	EMM177.0	E6	ST758	ERR227086
K6821	2001	1–4 years	swab	skin, soft tissue	EMM177.0	E6	ST758	ERR439226
K6847	2001	0–6 days	swab	skin, soft tissue	EMM65.5	E6	ST215	ERR449324
K6917	2001	7–27 days	swab	skin, soft tissue	EMM65.5	E6	ST215	ERR228688
K6932	2001	2–11 months	blood	skin, soft tissue	EMM217.0	D3	ST728	ERR449325
K7024	2001	0–6 days	swab	skin, soft tissue	EMM18.21	M18	ST402	ERR227092
K7087	2001	5 years and over	swab	skin, soft tissue	EMM11.0	E6	ST742	ERR227093
K7087	2001	5 years and over	swab	skin, soft tissue	EMM11.0	E6	ST742	ERR227094
K7113	2001	5 years and over	swab	skin, soft tissue	EMM11.0	E6	ST742	ERR227095
K7114	2001	5 years and over	swab	skin, soft tissue	STG866.1	NT	ST450	ERR228536
K7175	2001	1–4 years	blood	sepsis (no localizing source)	EMM165.0	E1	ST762	ERR228563
K7151	2001	7–27 days	swab	skin, soft tissue, pneumonia	EMM183.2	E3	ST761	ERR228538
K7219	2001	7–27 days	blood	sepsis (no localizing source)	EMM28.0	E4	ST763	ERR228620
K7275	2001	7–27 days	swab	skin, soft tissue	STG866.1	NT	ST450	ERR449326
K7367	2001	0–7 days	swab	skin, soft tissue	EMM55.0	M55	ST248	ERR439227
K7393	2001	1–4 years	blood	skin, soft tissue, pneumonia, stss	EMM63.5	E6	ST764	ERR228629
K7478	2001	1–4 years	blood	skin, soft tissue	EMM65.5	E6	ST215	ERR449327
K7498	2001	2–11 months	blood	sepsis (no localizing source)	EMM44.0	E3	ST178	ERR228614
K7541	2001	1–4 years	swab	skin, soft tissue	EMM81.2	E6	ST766	ERR228533
K7554	2001	7–28 days	blood	sepsis (no localizing source)	EMM90.5	E2	ST708	ERR228569
K7559	2001	7–28 days	blood	sepsis (no localizing source)	EMM110.0	E2	ST767	ERR228578
K7829	2001	5 years and over	swab	skin, soft tissue	EMM186.1	D4	ST262	ERR228526

Name	Year	Patient age category	Specimen type	Clinical presentation	*emm* sequence type	*emm*-cluster designation+	MLST	Genome Sequence Accession no.*
K7731	2001	7–28 days	blood	sepsis (no localizing source)	EMM100.2	D2	ST773	ERR228651
K7736	2001	5 years and over	swab	pneumonia, nephritis, stss, skin source	EMM165.0	E1	ST768	ERR228530
K7736	2001	5 years and over	blood	pneumonia, nephritis, stss	EMM19.10	M19	ST769	ERR228648
K7908	2001	5 years and over	swab	skin, soft tissue	EMM65.4	E6	ST129	ERR228527
K7792	2001	0–6 days	swab	skin, soft tissue, pneumonia	EMM43.1	D4	ST770	ERR228531
K7928	2001	neonate	swab	skin, soft tissue, stss	EMM4.5	E1	ST771	ERR228525
K7928	2001	neonate	swab	skin, soft tissue, stss	EMM4.5	E1	ST771	ERR449328
K8015	2001	5 years and over	swab	skin, soft tissue	EMM99.5	M95	ST781	ERR228608
K8057	2001	2–11 months	blood	pneumonia	EMM179.0	M179	ST619	ERR228643
K8396	2002	7–27 days	blood	sepsis (no localizing source)	EMM89.8	E4	ST772	ERR228612
K8460	2002	1–4 years	blood	sepsis (no localizing source)	EMM82.5	E3	ST257	ERR228600
K8492	2002	7–27 days	swab	skin, soft tissue	EMM100.2	D2	ST773	ERR228671
K8543	2002	2–11 months	blood	skin, soft tissue	EMM85.1	E6	ST774	ERR228641
K8728	2002	1–4 years	blood	sepsis (no localizing source)	EMM64.3	D4	ST223	ERR228690
K8744	2002	1–4 years	swab	skin, soft tissue and pneumonia	EMM8.3	E4	ST241	ERR228672
K8861	2002	7–27 days	blood	skin, soft tissue, stss	EMM44.0	E3	ST178	ERR228588
K8955	2002	7–27 days	swab	skin, soft tissue	EMM65.0	E6	ST778	ERR228542
K9037	2002	2–11 months	blood	skin, soft tissue	EMM239.1	A-C3	ST776	ERR228610
K9189	2002	2–11 months	blood	pneumonia	EMM182.1	E6	ST229	ERR228701
K9215	2002	28–60 days	blood	meningitis, pneumonia	EMM112.5	E4	ST777	ERR228632
K9333	2002	5 years and over	swab	skin, soft tissue	EMM90.5	E2	ST708	ERR228544
K9361	2002	2–11 months	blood	pneumonia	EMM44.0	E3	ST178	ERR228699
K9374	2002	28–60 days	swab	skin, soft tissue	EMM18.21	M18	ST221	ERR449329
K9400	2002	1–4 years	blood	skin, soft tissue	EMM90.5	E2	ST708	ERR449330
K9404	2002	1–4 years	blood	sepsis (no localizing source)	EMM90.5	E2	ST708	ERR439229
K9408	2002	2–11 months	swab	skin, soft tissue	EMM65.0	E6	ST778	ERR228551
K9429	2002	2–11 months	blood	pneumonia, stss	EMM95.0	E2	ST712	ERR439230
K9440	2002	28–60 days	blood	sepsis (no localizing source)	EMM99.5	E6	ST779	ERR228559
K9454	2002	2–11 months	blood	skin, soft tissue, stss	EMM50.3	E2	ST217	ERR228605
K37914	2009	5 years and over	urine	urinary tract infection	EMM119.2	D4	ST239	ERR228593
K9466	2002	2–11 months	blood	pneumonia	EMM78.5	E1	ST255	ERR439231
K9521	2002	1–4 years	blood	pneumonia	EMM84.1	E4	ST780	ERR228626
K9612	2002	1–4 years	blood	pneumonia	EMM99.5	E6	ST781	ERR228606

Name	Year	Patient age category	Specimen type	Clinical presentation	*emm* sequence type	*emm*-cluster designation+	MLST	Genome Sequence Accession no.*
K9612	2002	1–4 years	blood	pneumonia	EMM99.5	E6	ST781	ERR228642
K9679	2002	2–11 months	blood	pneumonia	EMM112.5	E4	ST777	ERR228596
K10040	2003	5 years and over	swab	skin, soft tissue	EMM22.5	E4	ST213	ERR228552
K10105	2003	5 years and over	swab	skin, soft tissue	EMM22.5	E4	ST213	ERR228553
K9887	2003	1–4 years	blood	skin, soft tissue	EMM119.2	D4	ST239	ERR449331
K9927	2003	1–4 years	swab	skin, soft tissue	EMM223.0	D4	ST613	ERR449332
K10016	2003	2–11 months	swab	skin, soft tissue and pneumonia	EMM103.0	E3	ST233	ERR228546
K10021	2003	5 years and over	swab	skin, soft tissue	EMM103.0	E3	ST233	ERR228547
K10167	2003	28–60 days	blood	pneumonia	EMM98.3	D4	ST136	ERR228625
K10213	2003	2–11 months	swab	skin, soft tissue	EMM169.1	E4	ST238	ERR228654
K10234	2003	2–11 months	blood	skin, soft tissue and pneumonia	EMM65.5	E6	ST215	ERR449333
K10238	2003	1–4 years	blood	nephritis	EMM218.1	M218	ST292	ERR228558
K10246	2003	1–4 years	swab	skin, soft tissue and pneumonia	EMM162.1	NT	ST412	ERR449334
K10311	2003	2–11 months	blood	skin, soft tissue	EMM50.3	E2	ST217	ERR228637
K10332	2003	7–28 days	swab	skin, soft tissue	EMM183.2	E3	ST761	ERR228480
K9340	2002	1–4 years	swab	skin, soft tissue	EMM18.21	M18	ST221	ERR439228
K10378	2003	2–11 months	blood	skin, soft tissue and pneumonia	EMM50.3	E2	ST217	ERR228599
K10474	2003	5 years and over	swab	skin, soft tissue	EMM118.2	E3	ST752	ERR228481
K10514	2003	2–11 months	blood	sepsis (no localizing source)	EMM77.0	E4	ST218	ERR228697
K10586	2003	1–4 years	swab	skin, soft tissue	EMM50.3	E2	ST217	ERR228482
K10676	2003	7–27 days	blood	sepsis (no localizing source)	EMM103.0	E3	ST233	ERR228623
K10697	2003	7–27 days	blood	sepsis (no localizing source)	EMM18.21	M18	ST221	ERR228570
K10712	2003	1–4 years	blood	skin, soft tissue	EMM183.2	E3	ST219	ERR228554
K10722	2003	2–11 months	swab	skin, soft tissue	EMM22.5	E4	ST213	ERR228483
K10812	2003	2–11 months	blood	pneumonia	EMM82.5	E4	ST257	ERR228638
K10987	2003	2–11 months	blood	pneumonia	EMM230.1	D4	ST755	ERR228557
K11116	2003	2–11 months	swab	skin, soft tissue	EMM18.21	M18	ST402	ERR228484
K11239	2003	1–4 years	swab	skin, soft tissue	EMM169.1	E4	ST238	ERR228485
K11243	2003	1–4 years	swab	skin, soft tissue	EMM114.5	E4	ST220	ERR228486
K11254	2003	0–6 days	blood	sepsis (no localizing source)	EMM18.21	M18	ST221	ERR228644
K11271	2003	1–4 years	blood	pneumonia	EMM25.4	E3	ST222	ERR228645
K11319	2003	1–4 years	swab	skin, soft tissue	EMM64.3	D4	ST223	ERR228487
K11464	2003	5 years and over	swab	skin, soft tissue	EMM99.5	E6	ST781	ERR228450
K11814	2004	5 years and over	swab	skin, soft tissue	EMM169.1	E4	ST226	ERR228451
K11898	2004	1–4 years	swab	skin, soft tissue	EMM111.2	M111	ST737	ERR227053
K12183	2004	7–27 days	blood	skin, soft tissue	EMM124.2	E4	ST231	ERR228590
K12363	2004	1–4 years	blood	skin, soft tissue	EMM79.5	E3	ST714	ERR439232
K12434	2004	5 years and over	swab	skin, soft tissue	EMM44.0	E3	ST178	ERR227050
K12452	2004	5 years and over	blood	skin, soft tissue	EMM183.2	E3	ST761	ERR439233

Name	Year	Patient age category	Specimen type	Clinical presentation	*emm* sequence type	*emm*-cluster designation+	MLST	Genome Sequence Accession no.*
K12473	2004	2–11 months	blood	skin, soft tissue	EMM55.0	M55	ST232	ERR227087
K13484	2004	0–6 days	blood	pneumonia	EMM119.2	D4	ST239	ERR228609
K12537	2004	1–6 months	CSF	meningitis, pneumonia	EMM103.0	E3	ST233	ERR449335
K12537	2004	1–6 months	blood	meningitis, pneumonia	EMM103.0	E3	ST233	ERR228693
K12554	2004	5 years and over	blood	sepsis (no localizing source)	EMM44.0	E3	ST178	ERR228635
K12614	2004	5 years and over	blood	skin, soft tissue	EMM179.0	M179	ST619	ERR228698
K12669	2004	7–27 days	blood	pneumonia	EMM183.2	E3	ST234	ERR228601
K13045	2004	2–11 months	blood	pneumonia	EMM192.0	D4	ST261	ERR228589
K13065	2004	5 years and over	swab	skin, soft tissue	EMM75.1	E6	ST578	ERR228488
K13107	2004	1–4 years	swab	skin, soft tissue	EMM183.2	E3	ST219	ERR228489
K13179	2004	7–28 days	blood	skin, soft tissue	STG7882.3	NT	ST235	ERR228691
K13190	2004	5 years and over	swab	skin, soft tissue	EMM39.4	A-C4	ST236	ERR228490
K13254	2004	1–4 years	blood	skin, soft tissue	EMM63.5	E6	ST764	ERR449336
K13372	2004	7–28 days	swab	skin, soft tissue, and pneumonia	EMM81.2	E6	ST766	ERR228491
K13389	2004	0–6 days	blood	sepsis (no localizing source)	EMM169.1	E4	ST238	ERR228667
K13389	2004	0–6 days	blood	sepsis (no localizing source)	EMM169.1	E4	ST238	ERR228700
K16898	2005	1–4 years	blood	skin, soft tissue	EMM112.5	E4	ST246	ERR227090
K13569	2004	2–11 months	blood	sepsis (no localizing source)	EMM63.5	E6	ST764	ERR439234
K13994	2005	2–11 months	blood	skin, soft tissue	EMM77.0	E4	ST747	ERR227056
K14810	2005	0–6 days	blood	skin, soft tissue	EMM18.21	M18	ST402	ERR228652
K16544	2005	5 years and over	swab	skin, soft tissue	EMM11.0	E6	ST250	ERR227057
K16587	2005	2–11 months	swab	skin, soft tissue	STG866.1	NT	ST450	ERR439235
K16727	2005	1–4 years	swab	skin, soft tissue	EMM65.0	E6	ST778	ERR227003
K16738	2005	7–27 days	blood	sepsis (no localizing source)	EMM64.3	D4	ST223	ERR228577
K16772	2005	7–27 days	blood	sepsis (no localizing source)	EMM89.8	E4	ST245	ERR228574
K16781	2005	7–27 days	swab	skin, soft tissue	STG866.1	NT	ST450	ERR227054
K16837	2005	1–4 years	swab	skin, soft tissue	EMM83.12	D4	ST393	ERR227055
K16849	2005	7–27 days	blood	sepsis (no localizing source)	EMM83.12	D4	ST393	ERR439236
K17011	2005	1–4 years	blood	skin, soft tissue	EMM79.5	E3	ST714	ERR449337
K17074	2005	5 years and over	swab	skin, soft tissue	EMM218.1	M218	ST292	ERR449338
K17097	2005	7–27 days	blood	pneumonia	EMM223.0	D4	ST536	ERR228631
K17300	2005	1–4 years	swab	skin, soft tissue	STG866.1	NT	ST450	ERR228453
K17276	2005	1–4 years	swab	skin, soft tissue	EMM92.0	E2	ST674	ERR228493
K17494	2005	2–11 months	blood	skin, soft tissue	EMM55.0	M55	ST248	ERR228561
K17716	2005	2–11 months	swab	skin, soft tissue and pneumonia	EMM19.10	M19	ST769	ERR228452
K17786	2006	28–60 days	blood	meningitis	EMM11.0	E6	ST251	ERR228597
K17786	2006	1–6 months	CSF	meningitis	EMM11.0	E6	ST250	ERR228584
K18724	2006	0–6 days	blood	pneumonia	EMM11.0	E6	ST250	ERR228568

Name	Year	Patient age category	Specimen type	Clinical presentation	*emm* sequence type	*emm*-cluster designation+	MLST	Genome Sequence Accession no.*
K19912	2006	5 years and over	swab	skin, soft tissue	EMM92.0	E2	ST674	ERR228509
K19083	2006	1–4 years	swab	skin, soft tissue	EMM56.0	D4	ST115	ERR227065
K19219	2006	1–4 years	swab	skin, soft tissue	EMM124.2	E4	ST231	ERR228454
K19188	2006	2–11 months	blood	skin, soft tissue	EMM8.3	E4	ST505	ERR449339
K19347	2006	5 years and over	swab	skin, soft tissue	stg1750.0	NT	ST258	ERR228503
K19376	2006	1–4 years	swab	skin, soft tissue	EMM75.1	E6	ST578	ERR228505
K19417	2006	5 years and over	swab	skin, soft tissue	EMM122.0	M122	ST200	ERR228522
K19464	2006	1–4 years	blood	skin, soft tissue and pneumonia	stg653.1	NT	ST254	ERR439237
K19639	2006	5 years and over	blood	endocarditis	EMM111.1	M111	ST496	ERR228560
K19669	2006	1–4 years	swab	skin, soft tissue	stg1750.0	NT	ST258	ERR228506
K19873	2006	7–27 days	CSF	meningitis, pneumonia	EMM78.5	E1	ST255	ERR228583
K19875	2006	7–27 days	swab	skin, soft tissue	EMM111.2	M111	ST256	ERR228508
K19952	2006	5 years and over	swab	skin, soft tissue, bone and joint	EMM92.0	E2	ST674	ERR228510
K19952	2006	5 years and over	swab	skin, soft tissue, bone and joint	EMM92.0	E2	ST674	ERR228511
K19961	2006	28–60 days	blood	pneumonia	EMM82.5	E3	ST257	ERR228615
K20001	2006	2–11 months	blood	sepsis (no localizing source)	EMM77.0	E4	ST747	ERR228611
K20201	2006	1–4 years	swab	skin, soft tissue	EMM44.0	E3	ST178	ERR228512
K22338	2006	5 years and over	swab	skin, soft tissue	STG866.1	NT	ST450	ERR227058
K20641	2006	2–11 months	swab	skin, soft tissue	EMM80.0	D4	ST701	ERR228472
K20653	2007	5 years and over	swab	skin, soft tissue	EMM209.0	E3	ST260	ERR228473
K20747	2007	1–4 years	swab	skin, soft tissue	EMM84.1	E4	ST259	ERR228476
K20746	2007	0–6 days	swab	skin, soft tissue	EMM97.1	D5	ST283	ERR228475
K20882	2007	5 years and over	swab	skin, soft tissue, bone and joint	EMM209.0	E3	ST260	ERR228477
K22813	2007	5 years and over	swab	skin, soft tissue and pneumonia	EMM121.0	D4	ST262	ERR227061
K20910	2007	0–6 days	swab	skin, soft tissue and pneumonia	EMM9.0	E3	ST447	ERR228478
K21246	2007	2–11 months	swab	skin, soft tissue	EMM77.0	E4	ST747	ERR228479
K21345	2007	28–60 days	swab	skin, soft tissue	STG866.1	NT	ST265	ERR227005
K21633	2007	28–60 days	blood	sepsis (no localizing source)	EMM15.1	E3	ST266	ERR228618
K21710	2007	0–6 days	swab	skin, soft tissue and pneumonia	EMM109.1	E4	ST718	ERR227064
K21771	2007	7–27 days	blood	sepsis (no localizing source), stss	EMM60.7	E1	ST700	ERR449341
K22633	2007	2–11 months	swab	skin, soft tissue	EMM89.8	E4	ST772	ERR227059
K22757	2007	1–4 years	swab	skin, soft tissue and pneumonia	EMM83.12	D4	ST393	ERR227060
K23180	2007	1–4 years	swab	skin, soft tissue	EMM44.0	E3	ST178	ERR227062
K23182	2007	1–4 years	blood	pneumonia	EMM63.5	E6	ST274	ERR228695
K23323	2007	1–4 years	swab	skin, soft tissue	EMM84.1	E4	ST259	ERR227063
K24357	2007	1–4 years	swab	skin, soft tissue	EMM209.0	E3	ST260	ERR439238
K23653	2007	1–4 years	swab	skin, soft tissue	EMM8.3	E4	ST241	ERR227004
K23617	2007	1–4 years	swab	skin, soft tissue	EMM48.0	E6	ST278	ERR227076
K23685	2007	2–11 months	swab	skin, soft tissue and pneumonia	EMM79.5	E3	ST714	ERR228494
K23745	2007	1–4 years	swab	skin, soft tissue	EMM60.7	E1	ST279	ERR228495

Name	Year	Patient age category	Specimen type	Clinical presentation	*emm* sequence type	*emm*-cluster designation+	MLST	Genome Sequence Accession no.*
K23866	2007	1–4 years	swab	skin, soft tissue and pneumonia	EMM82.5	E3	ST257	ERR228496
K23799	2007	5 years and over	swab	skin, soft tissue, bone and joint	EMM124.2	E4	ST280	ERR227007
K23890	2007	5 years and over	swab	skin, soft tissue	EMM97.1	D5	ST283	ERR228497
K24525	2007	5 years and over	swab	skin, soft tissue	EMM11.0	E6	ST404	ERR228501
K24190	2007	5 years and over	swab	skin, soft tissue	EMM28.0	E4	ST763	ERR228499
K24146	2007	1–4 years	swab	skin, soft tissue	EMM165.0	E1	ST768	ERR228498
K24601	2007	1–4 years	swab	skin, soft tissue	EMM89.8	E4	ST772	ERR228456
K24635	2007	1–4 years	swab	skin, soft tissue	EMM44.0	E3	ST178	ERR228457
K25147	2007	7–28 days	blood	pneumonia	EMM90.5	E2	ST708	ERR227051
K25325	2007	7–28 days	blood	pneumonia	EMM90.5	E2	ST708	ERR227073
K25713	2007	1–4 years	swab	skin, soft tissue	EMM124.2	E4	ST280	ERR228458
K26504	2007	5 years and over	swab	skin, soft tissue	EMM44.0	E3	ST178	ERR228459
K27345	2007	1–4 years	swab	skin, soft tissue	STG866.1	NT	ST450	ERR227032
K27345	2007	1–4 years	swab	skin, soft tissue	STG866.1	NT	ST450	ERR228520
K28044	2007	2–11 months	blood	pneumonia	EMM25.1	E3	ST323	ERR228521
K28044	2007	2–11 months	blood	pneumonia	EMM25.1	E3	ST323	ERR227033
K28162	2007	5 years and over	swab	skin, soft tissue	EMM83.12	D4	ST393	ERR228460
K29166	2008	1–4 years	blood	skin, soft tissue	EMM162.1	NT	ST412	ERR228461
K29527	2008	5 years and over	swab	skin, soft tissue	EMM4.5	E1	ST771	ERR228462
K29655	2008	5 years and over	swab	skin, soft tissue	EMM53.4	D4	ST460	ERR228463
K29743	2008	7–27 days	blood	pneumonia	EMM114.5	E4	ST220	ERR228464
K30067	2008	2–11 months	swab	skin, soft tissue	EMM73.0	E4	ST469	ERR228465
K30465	2008	2–11 months	swab	skin, soft tissue and pneumonia	EMM8.3	E4	ST505	ERR227030
K31028	2008	1–4 years	swab	skin, soft tissue	EMM93.6	D4	ST583	ERR228518
K31028	2008	1–4 years	swab	skin, soft tissue	EMM93.6	D4	ST613	ERR227052
K31063	2008	2–11 months	blood	skin, soft tissue and pneumonia	EMM209.0	E3	ST260	ERR228466
K38591	2009	5 years and over	swab	skin, soft tissue	EMM124.2	E4	ST231	ERR228441
K31539	2008	2–11 months	blood	sepsis (no localizing source)	EMM238.1	A-C3	ST713	ERR228467
K31611	2008	1–4 years	swab	skin, soft tissue	EMM93.6	D4	ST613	ERR228468
K32502	2008	2–11 months	swab	skin, soft tissue	EMM165.0	E1	ST768	ERR228469
K33951	2008	1–4 years	swab	skin, soft tissue	EMM80.0	D4	ST701	ERR228432
K33560	2008	neonate	CSF	skin, soft tissue and meningitis	EMM179.0	M179	ST619	ERR228470
K33560	2008	neonate	CSF	skin, soft tissue and meningitis	EMM179.0	M179	ST619	ERR228471
K33937	2008	1–4 years	swab	skin, soft tissue	EMM60.7	E1	ST700	ERR228429
K33937	2008	1–4 years	swab	skin, soft tissue	EMM60.7	E1	ST700	ERR228431
K33983	2008	0–6 days	swab	skin, soft tissue	EMM30.15	A-C2	ST537	ERR228433
K35129	2008	1–4 years	swab	skin, soft tissue	stg1750.0	NT	ST258	ERR228519
K35215	2008	5 years and over	swab	skin, soft tissue	STG866.1	NT	ST450	ERR439239
K35215	2008	5 years and over	swab	skin, soft tissue	STG866.1	NT	ST450	ERR228516
K35870	2008	5 years and over	swab	skin, soft tissue	EMM229.0	A-C4	ST703	ERR228434
K35909	2009	1–4 years	swab	skin, soft tissue	EMM75.1	E6	ST704	ERR228435
K36067	2009	1–4 years	aspirate	skin, soft tissue, bone and joint	EMM85.1	E6	ST774	ERR228436
K36294	2009	1–4 years	swab	skin, soft tissue	EMM49.9	E3	ST705	ERR228448

Name	Year	Patient age category	Specimen type	Clinical presentation	*emm* sequence type	*emm*-cluster designation+	MLST	Genome Sequence Accession no.*
K36347	2009	7–28 days	swab	skin, soft tissue	EMM65.0	E6	ST716	ERR228449
K36395	2009	1–4 years	swab	skin, soft tissue	EMM81.2	E6	ST766	ERR228514
K36535	2009	2–11 months	swab	skin, soft tissue and pneumonia	EMM100.2	D2	ST773	ERR227034
K36563	2009	2–11 months	blood	skin, soft tissue and pneumonia	EMM229.0	A-C4	ST703	ERR449343
K37164	2009	7–27 days	swab	skin, soft tissue	EMM224.1	D4	ST707	ERR228439
K37164	2009	7–27 days	swab	skin, soft tissue	EMM224.1	D4	ST707	ERR228446
K37287	2009	5 years and over	swab	skin, soft tissue	EMM55.0	M55	ST248	ERR228447
K37990	2009	2–11 months	blood	meningitis, pneumonia	EMM64.3	D4	ST223	ERR227010
K37990	2009	12–60 months	CSF	meningitis, pneumonia	EMM64.3	D4	ST223	ERR227011
K37698	2009	28–60 days	CSF	meningitis	EMM85.1	E6	ST709	ERR227027
K37698	2009	28–60 days	blood	meningitis	EMM85.1	E6	ST774	ERR227026
K37741	2009	7–27 days	blood	skin, soft tissue and pneumonia	EMM239.1	A-C3	ST776	ERR227035
K38181	2009	1–4 years	swab	skin, soft tissue	EMM56.0	D4	ST115	ERR227072
K38470	2009	5 years and over	swab	skin, soft tissue	EMM18.21	M18	ST402	ERR449344
K39244	2009	28–60 days	swab	skin, soft tissue and pneumonia	EMM80.0	D4	ST715	ERR439240
K40810	2010	2–11 months	swab	skin, soft tissue and pneumonia	EMM93.0	D4	ST10	ERR227074
K40818	2010	2–11 months	blood	skin, soft tissue and pneumonia	EMM112.5	E4	ST777	ERR227009
K41947	2010	1–4 years	swab	skin, soft tissue	EMM109.1	E4	ST718	ERR227075
K41948	2010	5 years and over	aspirate	skin, soft tissue, bone and joint	EMM208.0	D4	ST719	ERR228444
K42600	2010	1–4 years	swab	skin, soft tissue	EMM42.3	E6	ST721	ERR228445
K42771	2010	7–28 days	blood	pneumonia	EMM124.2	E4	ST231	ERR227015
K42952	2010	5 years and over	swab	skin, soft tissue	EMM111.2	M111	ST737	ERR449345
K43037	2010	1–4 years	blood	pneumonia	EMM44.0	E3	ST178	ERR449346
K43101	2010	1–4 years	aspirate	skin, soft tissue, bone and joint	EMM55.0	M55	ST248	ERR227044
K43304	2010	5 years and over	aspirate	skin, soft tissue, bone and joint	EMM18.21	M18	ST402	ERR227045
K44098	2010	5 years and over	blood	skin, soft tissue	EMM18.21	M18	ST402	ERR227042
K44582	2010	5 years and over	swab	skin, soft tissue	EMM57.0	M57	ST723	ERR227043
K44869	2010	5 years and over	swab	skin, soft tissue, bone and joint	STG866.1	NT	ST450	ERR227040
K44896	2010	5 years and over	blood	bone and joint	EMM99.5	E6	ST781	ERR227041
K45527	2010	1–4 years	blood	skin, soft tissue	EMM192.0	D4	ST724	ERR227038
K45900	2010	1–4 years	swab	skin, soft tissue	EMM118.2	E3	ST725	ERR227039
K46187	2010	5 years and over	swab	skin, soft tissue	EMM68.8	E2	ST726	ERR227024
K47020	2011	5 years and over	aspirate	skin, soft tissue, bone and joint	EMM80.0	D4	ST701	ERR227025
K47118	2011	1–4 years	swab	skin, soft tissue	EMM217.0	D3	ST728	ERR227022
K47483	2011	1–4 years	swab	skin, soft tissue	EMM225.0	D4	ST262	ERR439241
K47581	2011	1–4 years	swab	skin, soft tissue	EMM80.0	D4	ST729	ERR227020
K48083	2011	5 years and over	swab	skin, soft tissue	EMM179.0	M179	ST619	ERR227021
K48186	2011	1–4 years	swab	skin, soft tissue	stg1750.0	NT	ST731	ERR227018
K48650	2011	5 years and over		skin, soft tissue	EMM44.0	E3	ST178	ERR227019
K48817	2011	1–4 years	blood	sepsis (no localizing source)	EMM74.0	M74	ST120	ERR227049
K48807	2011	5 years and over	blood	necrotising fasciitis	EMM75.1	E6	ST704	ERR227048

Name	Year	Patient age category	Specimen type	Clinical presentation	*emm* sequence type	*emm*-cluster designation+	MLST	Genome Sequence Accession no.*
K48877	2011	1–4 years	aspirate	skin, soft tissue, bone and joint	EMM74.0	M74	ST732	ERR227046
K49294	2011	1–4 years	swab	skin, soft tissue	EMM90.5	E2	ST734	ERR227068
K49285	2011	5 years and over	swab	skin, soft tissue, bone and joint	EMM103.0	E3	ST733	ERR227047
K49551	2011	7–27 days	blood	pneumonia	EMM44.0	E3	ST178	ERR227069
K49882	2011	5 years and over	swab	skin, soft tissue, bone and joint	EMM111.2	M111	ST737	ERR227066
K50105	2011	1–4 years	swab	skin, soft tissue	EMM22.5	E4	ST213	ERR228517
K50105	2011	1–4 years	swab	skin, soft tissue	EMM22.5	E4	ST213	ERR227029
K50316	2011	1–4 years	swab	skin, soft tissue	EMM64.3	D4	ST223	ERR228523
K50593	2011	0–6 days	blood	pneumonia	EMM9.0	E3	ST740	ERR228507
K50658	2011	5 years and over	aspirate	skin, soft tissue, bone and joint	EMM9.0	E3	ST740	ERR228455
K50977	2011	5 years and over	blood	sepsis (no localizing source)	EMM179.0	M179	ST619	ERR449347
K51725	2011	1–4 years	swab	skin, soft tissue	EMM18.21	M18	ST402	ERR439242

*Short read sequence data available from the European Nucleotide Archive http://www.ebi.ac.uk/ena/
+NT: Non-typeable

Table 3. *emm*-clusters in children admitted to Kilifi County Hospital (1998-2011)

emm-cluster	N	%
E3	58	16.2
E4	56	15.7
E6	57	16.0
D4	51	14.3
NT*	26	7.3
E2	25	7.0
E1	16	4.5
M18	12	3.4
M179	9	2.5
M55	6	1.7
A-C3	5	1.4
M111	5	1.4
M74	4	1.1
A-C4	3	0.8
D2	5	1.4
D3	3	0.8
D5	3	0.8
M218	3	0.8
M122	2	0.6
M19	2	0.6
M95	2	0.6
A-C2	1	0.3
M26	1	0.3
M38	1	0.3
M57	1	0.3
Total	357	100

*NT stands for nontypeable

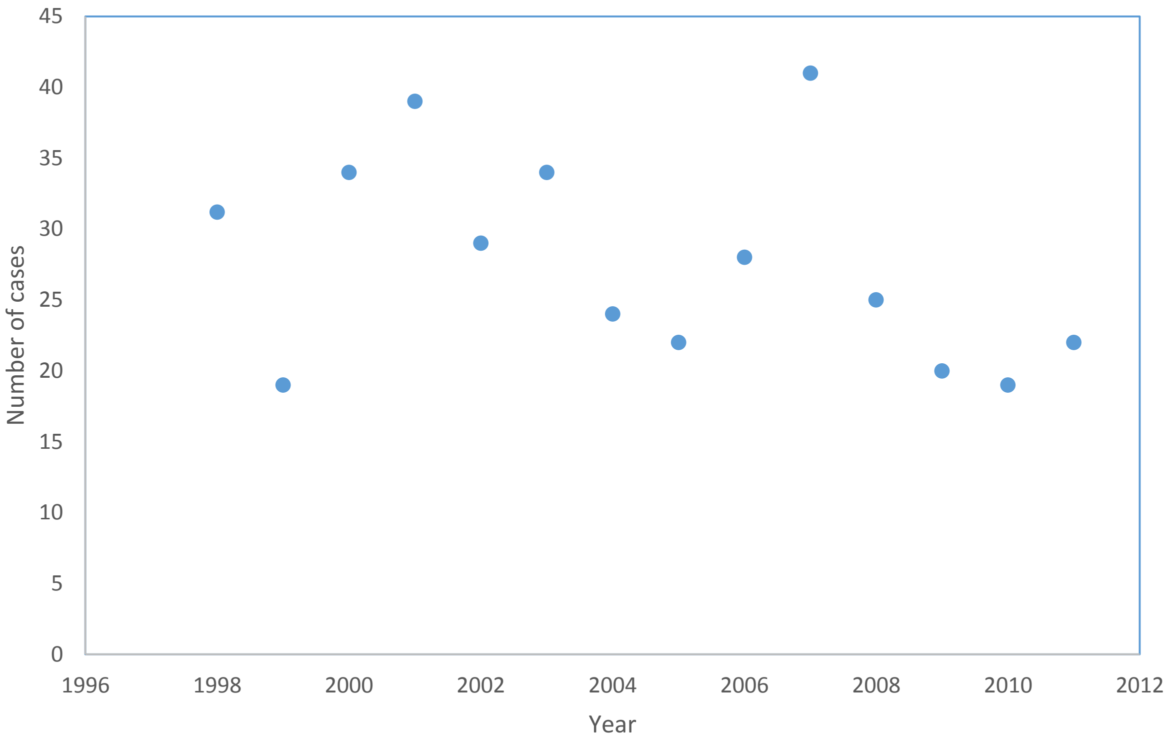

Technical Appendix Figure 1. Invasive group A *Streptococcus* cases in children admitted to Kilifi County Hospital, Kenya, during 1998–2011.

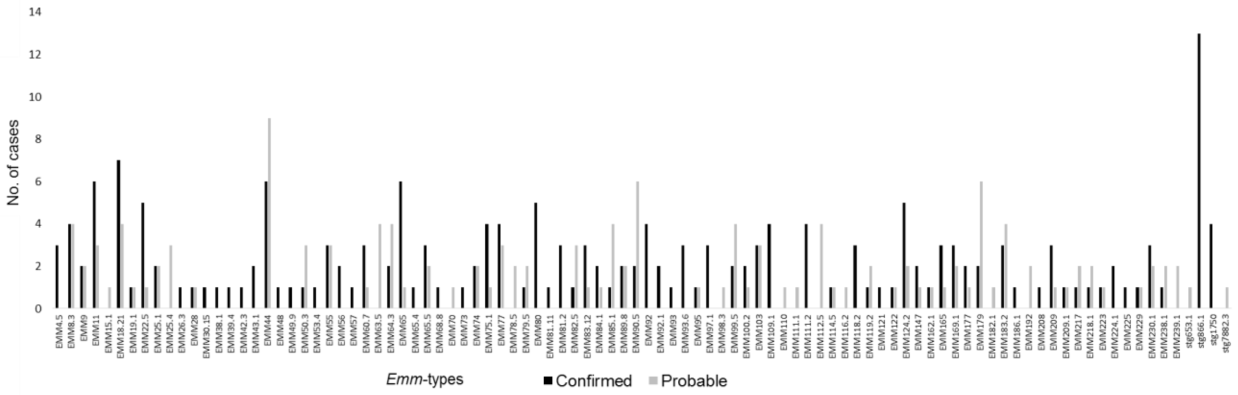

Technical Appendix Figure 2. *emm* types of group A *Streptococcus* (GAS) isolates from children admitted to Kilifi County Hospital, Kenya, with GAS disease during 1998–2011, by case definition.
